# Molecular barcoding of *Melissa officinalis* L. (badranjboye) in Iran and identification of adulteration in its medicinal services

**DOI:** 10.1186/s12870-022-03957-3

**Published:** 2022-12-15

**Authors:** Fahimeh Koohdar, Masoud Sheidai

**Affiliations:** Department of Plant Sciences and Biotechnology, Faculty of Life Sciences and Biotechnology, Shahid Beheshti University, Tehran, Iran

**Keywords:** *Melissa officinalis*, DNA barcoding, ITS, Chloroplast DNA

## Abstract

Medicinal plants are an important source for treatment of diseases in many countries. Today, controlling the quality of the products of medicinal plants is an important task. Customer health may be in danger due to the fraud and misconduct by the sales associates in the sales centers. *Melissa officinalis* L. (Lamiaceae) is an important medicinal plant used for treatment of several diseases. In Iran, the species of *Dracocephalum*, *Hymencrater*, *Nepeta* and *Stachys* are mistakenly sold under the name of Badranjboye that have different pharmaceutical properties. For avoiding this mistake, this investigation was performed with the following aims: 1) Checking for the cheating and identifying the Badranjboye in the Iran’s market of medicinal plants, 2) Providing the molecular barcode for the medicinal species of *Melissa*. For this purpose, Market-sold plant samples (leaves) and original reference plant species were compared by morphology, odor as well as Internal transcribed spacer (ITS) and chloroplast DNA ((*psbA*–*trnH*) and (*trnL*–*trnF*)) sequences. Various molecular analyses, such as sequencing, determination of genetic distance, and construction of phylogenetic tree were performed. These reports have shown that internal transcribed spacer (ITS) and chloroplast DNA (*psbA*–*trnH*) sequences are an efficient molecular marker to produce barcode gap and differentiating *Melissa officinalis* from other species.

## Introduction

Nowadays, identifying and applying the proper curing approaches versus harms threatening the human wellbeing including the presence of harmful chemicals and microorganisms in the food, water contamination, and illnesses is of great importance [1]. Traditional medicine is an important and historical treatment option in many countries including Iran [2].

Adulteration is referred to the deliberate replacement or addition of another plant or external material in order to increase the weight, power, or decrease the cost of the product, generally considered as deliberate action. Thus, the forgery could occur due to the lack of knowledge about the original plant, the similarity in the name, morphology, and the aroma of species associated with low-cost plant material, so the wrong kinds should be identified from the beginning because of improper use of medicinal plants can cause irreparable damage to the body [3].

In this regard, in a study conducted in China, 1-hundred female patients were detected to have renal disease because of incorrect application of *Stephania tetrandra* S. Moore instead *Aristolochia fangchi* Y.C.Wu ex L.D.Chow & S.M.Hwang due to the similarity in the name and morphology of it. Other similar cases can mention the skin of the *Cinnamomum verum* J. Presl that causes poisoning because of mixing with toxic *Cinnamomum cassia* (L.) J.Presl species [1].

The morphological, anatomical, chemical, and molecular techniques are used as a general method for identification of the plants. Therefore, the traditional classification requires the expertise of the professionals with professional experience. In some cases, it may be difficult for the experts to identify the samples without diagnostic parts [3–5].

Microscopic mass spectrometry and more recently, DNA barcoding, genomics, proteomics, and metabolomics techniques have been used to differentiate the original medicinal plants from wrong ones [5, 6].

DNA barcoding is a sequence of DNA that can be useful in the fast and precise identification of species. It has been applied for identifying the medication-related herbs and has differentiated the real and authentic goods from their counterfeit ones [7, 8]. The main principles of DNA barcoding are standardization, minimalism, and scalability, which means selection one or a few standard loci that can be sequenced routinely and reliably in very large and diverse sample sets, and obtaining a reliable and conveniently comparable data to differentiate the species in question from one another [9]. These methods provide suitable tools for analysis of the medicinal plants in the markets, requiring precise identification of the plant species, such as sample sequences, comparing the obtained sequence with recorded ones in the reference database, and various nucleotide sequences to differentiate the similar species [6, 10].

Identification of the medicinal plants based on the DNA sequence, multiple, and its variation with sister species is currently used. Different researches resulted in general agreement that several different marker combinations produce equivalent performance, and that none of the proposed barcodes is perfect in every respect [11]. Utilizing a multiple approach for a better species differentiation has been suggested by several authors (see for example, Fazekas [12]).

In most of the studies, researchers use of a common, easily amplified and aligned region such as *rbcL*, *trn L*-*F* spacer regions, *mat K*, *trnHG-psbA*, *nrITS1*, *nrITS2* or the full *ITS1*–*5.8S*-*ITS2* (*nrITS*), as suggested by the CBOL Plant Working Group and BOLD [13, 14].

In Iran, there are around 8000 species of plants, among which 2300 species are aromatic and medicinal, and 450 species are sold in the traditional herbal shop [2].

*Melissa officinalis* L. is one of the medicinal species known as Badranjboye in Iran. This species is well known as a medicinal plant and is used by the people in a variety of forms such as essential oils, oily extract, oil, and infusion and has various properties including the treatment of stomach disorders with neural origin. Badranjboye as an essential drug has been prescribed by Avicenna for enhancement of the heart and the expansion of the soul. This plant has been used as an antidepressant until the seventeenth century [15–18].

In Iran, *Dracocephalum* L., *Hymenocrater* Fisch. & C.A.Mey.*, Nepeta* L. and *Stachys* L. are wrongly named as Badranjboye. Thus, none of these species will have the impacts of the aforementioned plant if they are applied. They are different such that, Badranjboye possesses heart - formed leaves, whilst *Dracocephalum moldavica* L. possesses lengthy leaves with a longitude of 3–5 cm and width of 1–5 cm. Moreover, it is different from *Hymenocrater* having odorless leaves [19].

Considering that the identification of plants is sometimes not possible through their appearance due to their powdery form, in this study, the following objectives will be followed: 1) Determining the integrity of medicinal plants in the country’s pharmaceutical market and detecting any fraud through molecular approaches and 2) Developing a molecular barcode to identify the species *Melissa officinalis* from other items identified as Badranjboye.

## Material and methods

For determining the DNA barcode, leaves of the medicinal plants identified under the name of Badranjboye were collected from 22 traditional herbal shops in eight provinces in Iran. Voucher specimens are deposited in Herbarium of Shahid Beheshti University (HSBU) (Table 1). For determining the appropriate parts for the barcodes, the sequences were recorded at the National Center for Biotechnology Information (NCBI) (https://www.ncbi.nlm.nih.gov/). for ITS and chloroplast region (*trnH- psbA*, *trnL-trnF*) of *Melissa*, *Dracocephalum*, and *Hymenocrater* were first obtained (Table 2).

**Table 1:** The locality of medicinal plants as badranjboye

Number	Province	voucher number	Number	Province	voucher number
1	Tehran	HSBU2016150	12	Mazandaran, Babolsar	HSBU2016161
2	Tehran	HSBU2016151	13	Mashhad, Neyshabur	HSBU2016162
3	Alborz	HSBU2016152	14	Mashhad, Sabzevar	HSBU2016163
4	Alborz	HSBU2016153	15	Mashhad, Sabzevar	HSBU2016164
5	Alborz	HSBU2016154	16	Mashhad, Sabzevar	HSBU2016165
6	Alborz	HSBU2016155	17	Ardabil	HSBU2016166
7	Alborz	HSBU2016156	18	Tabriz	HSBU2016167
8	Shiraz	HSBU2016157	19	Lorestan	HSBU2016168
9	Shiraz	HSBU2016158	20	Lorestan	HSBU2016169
10	Shiraz	HSBU2016159	21	Isfahan	HSBU2016170
11	Mazandaran, Amol	HSBU2016160	22	Isfahan	HSBU2016171

**Table 2:** The spices names and gene bank number of taxa in ITS and cp-DNA studies

NO	Spices names and Gene bank numbers of ITS marker	NO	Spices names and Gene bank numbers of trnH-psbA	NO	Spices names and Gene bank numbers of trnL- trnF
1	*Melissa officinalis* (EU796895.1)	1	*Melissa axillaris* (KY197901.1)	1	*Melissa officinalis* (JF301386.1)
2	*Melissa officinalis* (DQ189090.1)	2	*Melissa officinalis* (KC584964.1)	2	*Dracocephalum moldavica* (AY506625.1)
3	*Melissa officinalis* (MK425905.1)	3	*Melissa officinalis* (HQ902824.1)	3	*Dracocephalum kotschyi* (KX641651.1)
4	*Melissa officinalis* (DQ667291.1)	4	*Melissa officinalis* (KP643311.1)	4	*Melissa axillaris* (JQ669051.1)
5	*Melissa axillaris* (KM886748.1)	5	*Melissa officinalis* (MH781964.1)	5	*Hymenocrater platystegius* (LC316173.1)
6	*Melissa axillaris* (JQ669114.1)	6	*Melissa officinalis* (LS999865.1)	6	*Hymenocrater bituminosus* (JQ669045.1)
7	*Melissa axillaris* (MH808584.1)	7	*Dracocephalum moldavica* (MF371112.1)	7	*Hymenocrater calycinus* (LC316155.1)
8	*Dracocephalum moldavica* L. (AY506659.1)	8	*Dracocephalum integrifolium* (MF371110.1)	8	*Melissa officinalis* (AJ505529.1)
9	*Dracocephalum kotschyi* Boiss*.* (AJ420998.1)	9	*Hymenocrater bituminosus* (MH175478.1)	9	*Melissa axillaris* (KM886646.1)
10	*Hymenocrater bituminosus* Fisch. & C.A. Mey. (JQ669105.1)	10	*Hymenocrater incanus* (MH175477.1)		
11	*Hymenocrater elegans* Bunge. J Essent. (LC316148.1)				
12	*Hymenocrater platystegius* Rech. f., H. (LC316140.1)				
13	*Hymenocrater calycinus* (Boiss.) Benth. (LC316147.1)				

### DNA extraction and amplification

For traditional herbal shops samples, genomic DNA was extracted using the (Cetyl Trimethylammonium Bromide) CTAB by the activated charcoal protocol. The quality and quantity of the extracted DNA were assessed by running the electrophoresis on 0.8% agarose gel [20]. ITS region (ITS1, 5.8S, ITS2) was amplified with primer ITS1 (5′-TCCGTAGGTGAACCTGCGG-3’and primer ITS2 (5′ GCTGCGTTCTTCATCGATGC-3′) [21]. The intergenic spacer of chloroplast genome *psbA-trnHG* was sequenced with universal primers following the methodology of Shaw [22] and Timmer [23]. The *psbA-trnHG* forward primer was (t*rnHG*) 5′ CGCGCATGGTGGATTCACAATCC − 3′ and, the reverse primer was (psbA) 5′- GTTATGCATGAACGTAATGCTC − 3′.

The PCR reaction mixture consisted of 20 ng genomic DNA and 3 U of Taq DNA polymerase (Bioron, Germany); 50 mM KCl; 10 mM Tris-HCl buffer at pH 8; 1.5 mM MgCl2; 0.2 mM of each dNTP (Bioron, Germany); 0.2 l M of each primer in a total volume of 25 pi.

DNA amplification was performed on a BIO RAD (T100 Thermal cycler) with the following program: 5 min at 94 C°, 35 cycles of 1 min at 94 C°, 1 min at 50 C° in trnH-psbA and 54C° at ITS primers and 1 min at 72 C and a final cycle of 7 min at 72 C°.

The PCR amplified products were separated by electrophoresis on 2% agarose gels (Merck). The gels were stained with ethidium bromide and visualized under UV light or silver stained for added sensitivity. Fragment size was estimated by using a 100 base pair (bp) molecular size ladder (Fermentas, Germany).

### Data analyses

ITS and chloroplast (*trnH*-*psbA*, *trnL*-*F*) sequences obtained from the NCBI (Table 2) were analyzed after alignment and curation using molecular evolutionary genetics analysis (MEGA) ver. 7 software [24]. Maximum likelihood, maximum parsimony, (Neighbor-Joining) NJ and (Unweighted Pair Group Method with Arithmetic mean) UPGMA tree were plotted to show the delimitation between the genera and their species. After clustering and measuring the distance between *Dracocephalum*, *Hymenocrater, and Melissa* species based on ITS, *trnH*-*psbA* and *trnL*-*F* markers, it was concluded that ITS and *trnH*-*psbA* are the most appropriate markers for the *Melissa* barcode. Therefore, the Iran’s market samples were amplified by these markers.

The unknown barcodes from Iran’s market leaves were basically identified by the Basic Local Alignment Search Tool (BLAST) data with a minimum BLAST identity cut off of 97% for a top match. These results were verified by clustering and phylogenetic analyses where the branches of unknown specimens were compared with the sequences of reference species using the MEGA software ver. 7 [23].

Two different approaches were used for barcode gap analysis. Firstly, aligned sequences of both reference plants (*Melissa officinalis*) and potential adulterants (*Dracocephalum*, *Hymenocrater, and* the other *Melissa* species) were compared in order to identify barcodes and differentiating sequences between reference plants and the potential adulterants (commonly mixed products), as well as common sequences in the reference plant that differ with the adulterant plant species were searched.

Secondly, the species discrimination power of the investigated loci was also assessed using the genetic distance approach, to evaluate whether the amount of variation displayed was sufficient to discriminate sister species without affecting their correct assignation through intra-specific variation. This approach is the basis of the “barcoding gap” definition, i.e. the assumption that the amount of sequence divergence within species is smaller than that between species. For this purpose, Kimura 2-parameter model of genetic distance in different sequences was used within and among the congeneric species by the molecular evolutionary genetic analysis using the MEGA7 software [2, 24].

The true medicinal plant species presenting a minimum interspecific distance value higher than their maximum intraspecific distance were considered successfully discriminated from potential adulterant plant species [25].

## Results

### Investigation of delimitation between medicinal genera and species using ITS marker

**Fig. 1:**
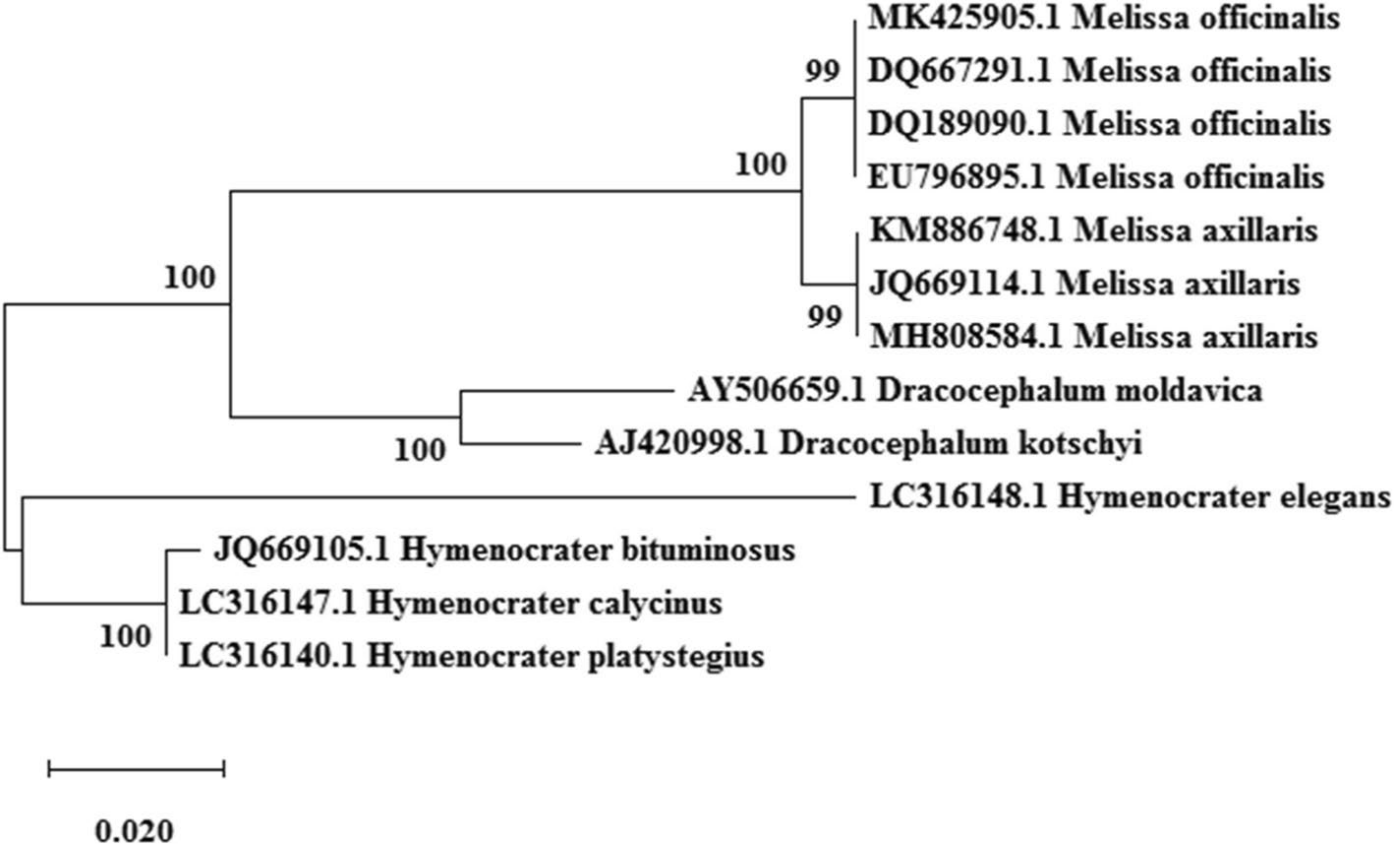
Maximum likelihood phylogenetic tree of NCBI taxa based on ITS Sequences

**Fig. 2:**
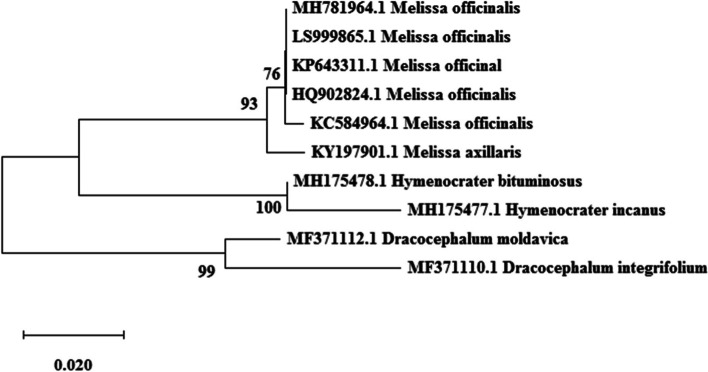
Maximum likelihood phylogenetic tree of NCBI taxa based on trnH-psbA Sequences

**Fig. 3:**
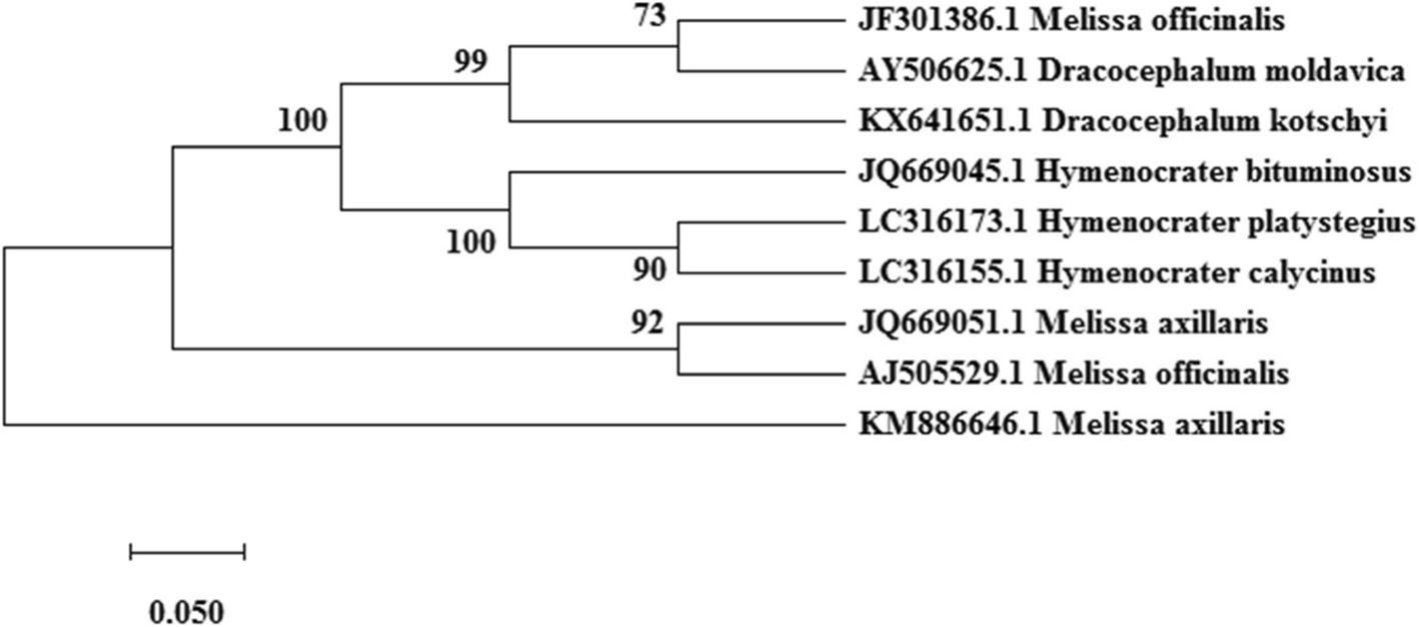
Maximum likelihood phylogenetic tree of NCBI taxa based on trnL-F Sequences

ITS, *trnH*-*psbA*, and *trnL*-*F* sequences (Table 2) were analyzed after alignment and curation using the MEGA software ver. 7. In the maximum likelihood plot developed based on ITS and and *trnH*-*psbA* Sequences (Figs. 1 and 2), *Melissa officinalis* samples were separated from *Hymenocrater*, *Dracocephalum* and *Melissa axillaris* (Benth.) Bakh.f. but these genera could not be separated from the others based on *trnL*-*F* sequences (Fig. 3). Therefore, this region was not suitable for DNA barcoding in *Melissa officinalis.*

Based on Kimura 2-parameters genetic distance in ITS and *trnH*-*psbA* regions, there was no genetic distance within *Melissa officinalis* (0.00), This result revealed the absence of sequence variability within ITS and *trnH*-*psbA* sequences within *Melissa officinalis* (high uniformity). A high value of genetic distance occurred between *Melissa officinalis* with other species (*Melissa axillar*) and genera (*Dracocephalum* and *Hymenocrater*) (0.08 to 0.18). Kimura 2-parameters genetic distance based on trnL-F region showed a high value of genetic distance within *Melissa officinalis*.

**Fig. 4:**
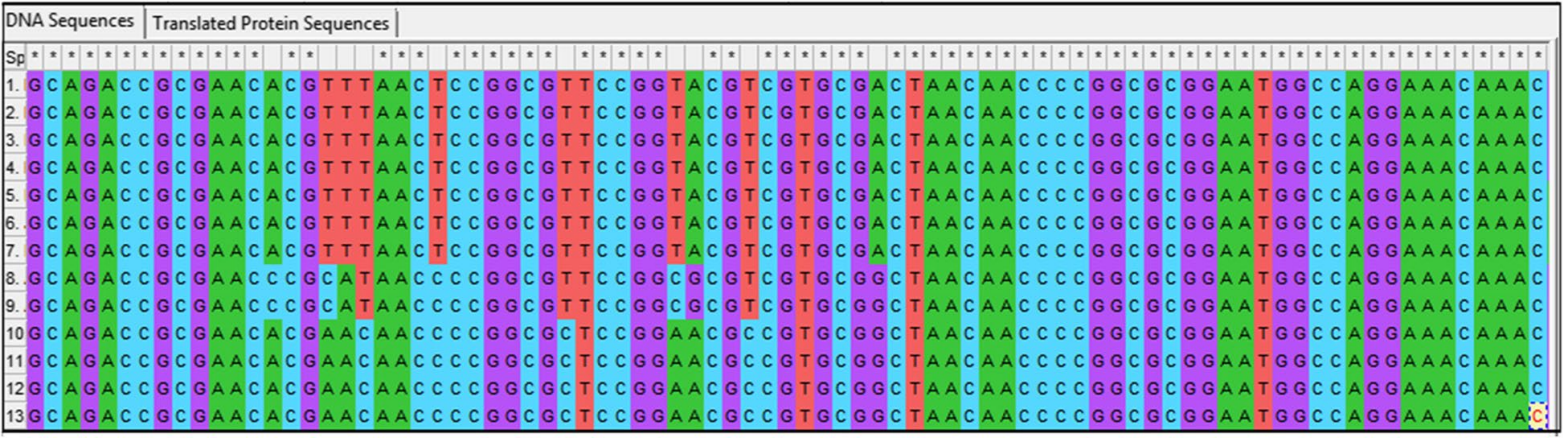
Barcode gaps in ITS sequences of *Melissa officinalis* (The numbers are according to Table 2)

**Fig. 5:**
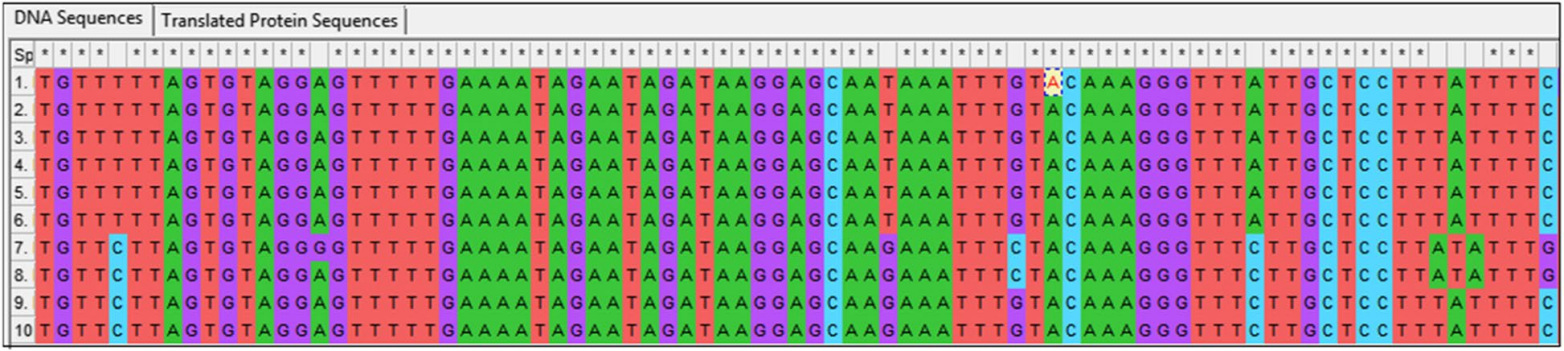
Barcode gaps in trnH-psbA sequences of *Melissa officinalis* (The numbers are according to Table 2)

Common and specific sequences were evaluated in the ITS and *trnH*-*psbA* genetic regions. It was attempted to create the barcode gaps between the studied species and genera. Accordingly, in ITS region, six nucleotides with numbers of (117–131–227-522-650-655) were identified as barcode gap for *Melissa officinalis* and *Melissa axillaris* while *trnH*-*psbA* region could only identify two barcodes to differentiate. Thirty nucleotides were used as a barcode gap for separation of *Melissa* genus from *Hymenocrater* and *Dracocephalum* in both regions. ITS and *trnH*-*psbA* region can be considered as a barcode gap in *Melissa officinalis* (Figs. 4 and 5). Therefore, it is suggested to use the ITS and trnH-psbA sequences as barcode gaps for barcoding gap and differentiating between *Melissa officinalis* and other species and genera*.*

### Morphology and odor of the medicinal products

Morphologically, the leaves of the samples sold in the market were similar to *Melissa*, *Dracocephalum*, and *Hymenocrater*. It should be noted that, there were leaves belonging to the Boraginaceae and Poaceae families in the collected specimens. Two samples were in powder form and could not be identified by their leaves. For checking the aroma in the samples, we used Sheidai [2]. *Melissa officinalis* smells like lemon while other genus does not have this smell. The studied samples were mostly odorless and occasionally had a smell of lemon (Table 3).

**Table 3:** Medicinal plant products studied their odor and leaf morphological resemblance

NO	Province	Leaf shape	Odor	NO	Province	Leaf shape	Odor
1	Tehran	*Asperugo*	Nothing	12	Mazandaran, Babolsar	*Dracocephalum and Hymenocrater*	Lemon
2	Tehran	*Asperugo*	Nothing	13	Mashhad, Neyshabur	*Dracocephalum and Hymenocrater*	Lemon
3	Alborz	Nothing	Nothing	14	Mashhad, Sabzevar	*Dracocephalum and Hymenocrater*	Lemon
4	Alborz	*Asperugo*	Nothing	15	Mashhad, Sabzevar	*Dracocephalum and Hymenocrater*	Lemon
5	Alborz	*Dracocephalum and Hymenocrater*	Lemon	16	Mashhad, Sabzevar	*Dracocephalum and Hymenocrater*	Nothing
6	Alborz	Nothing	Lemon	17	Ardabil	Nothing	Nothing
7	Alborz	*Mellisa* and *Hymenocrater*	Lemon	18	Tabriz	Nothing	Nothing
8	Shiraz	*Mellisa* and *Hymenocrater*	Lemon	19	Lorestan	*Mellisa* and *Hymenocrater*	Lemon
9	Shiraz	*Mellisa* and *Hymenocrater*	Lemon	20	Lorestan	*Mellisa* and *Hymenocrater*	Lemon
10	Shiraz	*Mellisa* and *Hymenocrater*	Lemon	21	Isfahan	*Dracocephalum and Hymenocrater*	Lemon
11	Mazandaran, Amol	*Dracocephalum and Hymenocrater*	Nothing	22	Isfahan	*Dracocephalum and Hymenocrater*	Lemon

### Blast results of the market products/samples studied based on ITS and trnH-psbA regions

**Fig. 6:**
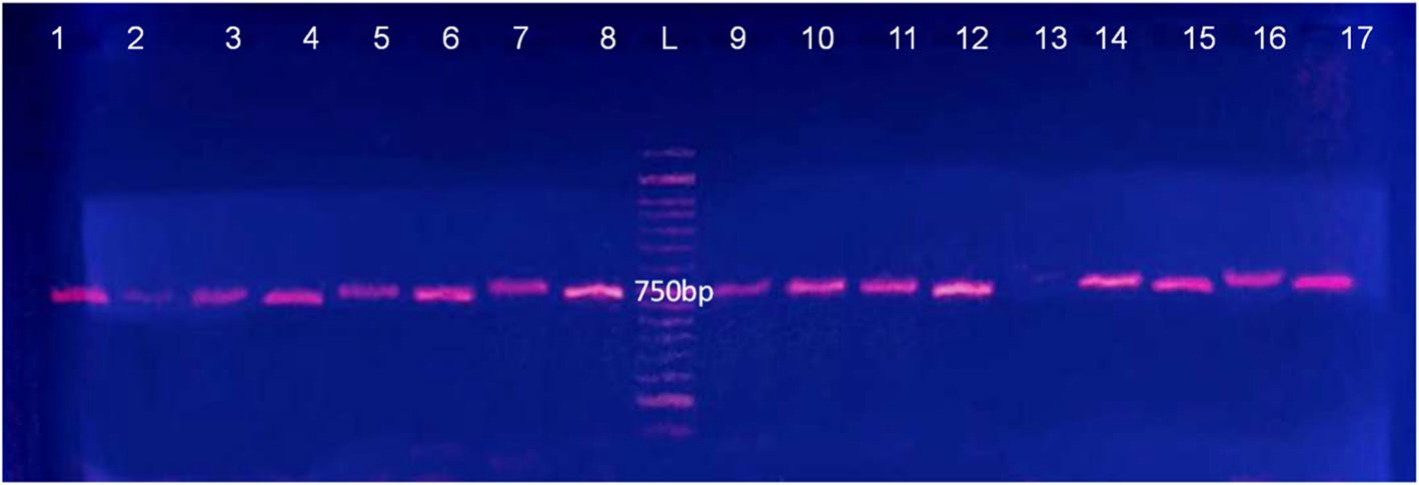
PCR gel of some studied *Melissa officinalis* based on ITS marker (The numbers are according to Table 1)

Sequencing of *ITS and trnH-psbA regions*, produced about 750 nucleotides. PCR gel photos and BLAST analysis of the market products/samples studied based on ITS and *trnH-psbA* regions showed similar results; therefore, only ITS results are presented in Table 4 and Fig. 6. The samples sold in the market as Badranjboye (*Melissa officinalis*) showed sequence similarity with various species and genera like *Asperugo procumbens* L. and *Mertensia virginica* (L.) Pers. ex Link of Boraginaceae family, *Trigonella foenum-graecum* L. and *Melilotus officinalis* (L.) Pall. of Fabaceae family, *Hymenocrater* and *Dracocephalum* of Lamiaceae family. Only seven samples in 22 samples were similar to *Melissa officinalis*.

**Table 4:** The BLAST results of the sample products

Number of Medicinal plant based on Table 1	Blast of species of NCBI	Accession	Identity
1	*Asperugo procumbens*	JQ388496.1	96.57%
	*Mertensia virginica*	JQ388507.1	91.78%
2	*Asperugo procumbens*	JQ388497.1	98.97%
	*Mertensia alpina*	JQ388507.1	94.26%
3	*Asperugo procumbens*	JQ388496.1	99.12%
	*Mertensia alpina*	JQ388507.1	94.69%
4	*Asperugo procumbens*	JQ388497.1	99.56%
	*Mertensia alpina*	JQ388507.1	94.92%
5	*Hymenocrater sessilifolius*	LC316142.1	85.25%
	*Hymenocrater calycinus*	LC316147.1	85.04%
	*Hymenocrater bituminosus*	LC316144.1	85.04%
	*Hymenocrater platystegius*	LC316140.1	85.04%
	*Hymenocrater bituminosus*	JQ669105.1	84.63%
6	*Melissa officinalis*	KJ584249.1	100.00%
7	*Melissa officinalis*	AY506650.1	98.75%
8	*Melissa officinalis*	AY506650.1	99%
	*Melissa officinalis*	KJ584249.1	98.75%
	*Melissa officinalis*	EU796895.1	96%
	*Melissa officinalis*	DQ667291.1	95%
9	*Melissa officinalis*	EU796895.1	99%
	*Melissa officinalis*	DQ667291.1	96%
	*Melissa officinalis*	DQ189090.1	95%
	*Melissa officinalis*	JF301353.1	95%
10	*Melissa officinalis*	DQ667291.1	99%
	*Melissa officinalis*	DQ189090.1	96%
	*Melissa officinalis*	JF301353.1	95%
	*Melissa officinalis*	KY072952.1	95%
11	*Hymenocrater bituminosus*	LC316144.1	92%
	*Hymenocrater platystegius*	LC316140.1	92%
	*Hymenocrater bituminosus*	JQ669105.1	94%
	*Hymenocrater sessilifolius*	LC316142.1	92%
12	*Hymenocrater platystegius*	LC316140.1	92%
	*Hymenocrater bituminosus*	JQ669105.1	94%
	*Hymenocrater sessilifolius*	LC316142.1	92%
	*Hymenocrater calycinus*	LC316147.1	92%
13	*Dracocephalum moldavica*	AY506659.1	95.45%
	*Dracocephalum bullatum*	JQ669096.1	95.45%
	*Dracocephalum parviflorum*	JQ669097.1	95.33%
14	*Hymenocrater calycinus*	LC316147.1	85.04%
	*Hymenocrater bituminosus*	LC316144.1	85.04%
	*Hymenocrater platystegius*	LC316140.1	85.04%
	*Hymenocrater bituminosus*	JQ669105.1	84.63%
15	*Hymenocrater sessilifolius*	LC316142.1	92%
	*Hymenocrater calycinus*	LC316147.1	92%
	*Hymenocrater bituminosus*	LC316144.1	92%
	*Hymenocrater platystegius*	LC316140.1	92%
16	*Dracocephalum moldavica*	AY506659.1	91%
	*Dracocephalum moldavica*	MH710906.1	90%
	*Dracocephalum bullatum*	JQ669096.1	90%
	*Dracocephalum kotschyi*	AJ420998.1	90%
17	*Trigonella foenum-graecum*	DQ312196.1	99.72%
	*Melilotus officinalis*	DQ311985.1	96.47%
18	*Trigonella foenum-graecum*	DQ312196.1	99.72%
	*Melilotus officinalis*	DQ311985.1	96.47%
19	*Melissa officinalis*	KY072952.1	98%
	*Melissa officinalis*	AY506650.1	97%
	*Melissa axillaris*	JQ669114.1	97%
	*Melissa axillaris*	KM886748.1	96%
20	*Melissa officinalis*	KY072952.1	98%
	*Melissa officinalis*	AY506650.1	97%
	*Melissa axillaris*	JQ669114.1	97%
	*Melissa axillaris*	KM886748.1	96%
21	*Hymenocrater calycinus*	LC316147.1	92%
	*Hymenocrater bituminosus*	LC316144.1	92%
	*Hymenocrater platystegius*	LC316140.1	92%
	*Hymenocrater bituminosus*	JQ669105.1	90%
22	*Hymenocrater calycinus*	LC316147.1	92%
	*Hymenocrater bituminosus*	LC316144.1	92%
	*Hymenocrater platystegius*	LC316140.1	92%
	*Hymenocrater bituminosus*	JQ669105.1	90%

### Genetic distance results within and between market samples and the reference species based on ITS and trnH-psbA regions

Kimura 2-parameter model of genetic distance was provided to determine the genetic distance both within the studied market samples and between the samples and the reference species based on ITS and *trnH*-*psbA* regions. Results showed that, the market samples have high genetic distance with each other for example, the genetic distance between the samples obtained from Shiraz (No 2 based on Table 1) and samples from Isfahan and Ardabil provinces (No 2 based on Table 1) was from 0.6 to 0.10, while they were highly similar with the samples having the highest percentage of similarity in the BLAST (Table 4) (0.00–0.02). This gap indicates that the samples are not identical in the market implying the fraud in their sales.

### Phylogenetic analyses of the market products/samples studied based on ITS and trnH-psbA regions

**Fig. 7:**
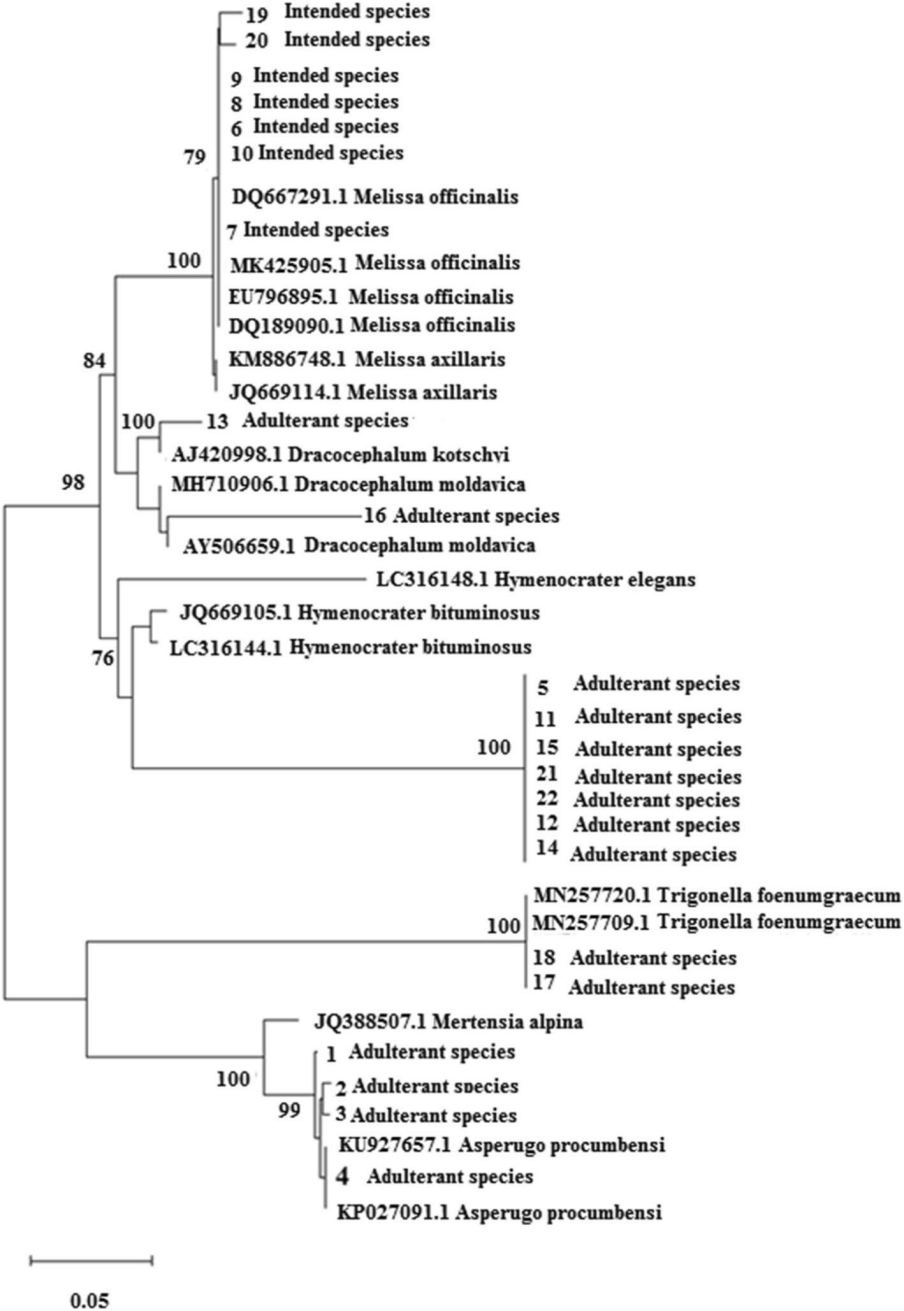
Maximum likelihood tree of the studied market products (1 to 22 based on Table 1) and reference species identified by BLAST query, based on ITS sequences. (Numbers above branches are bootstrap value)

**Fig. 8:**
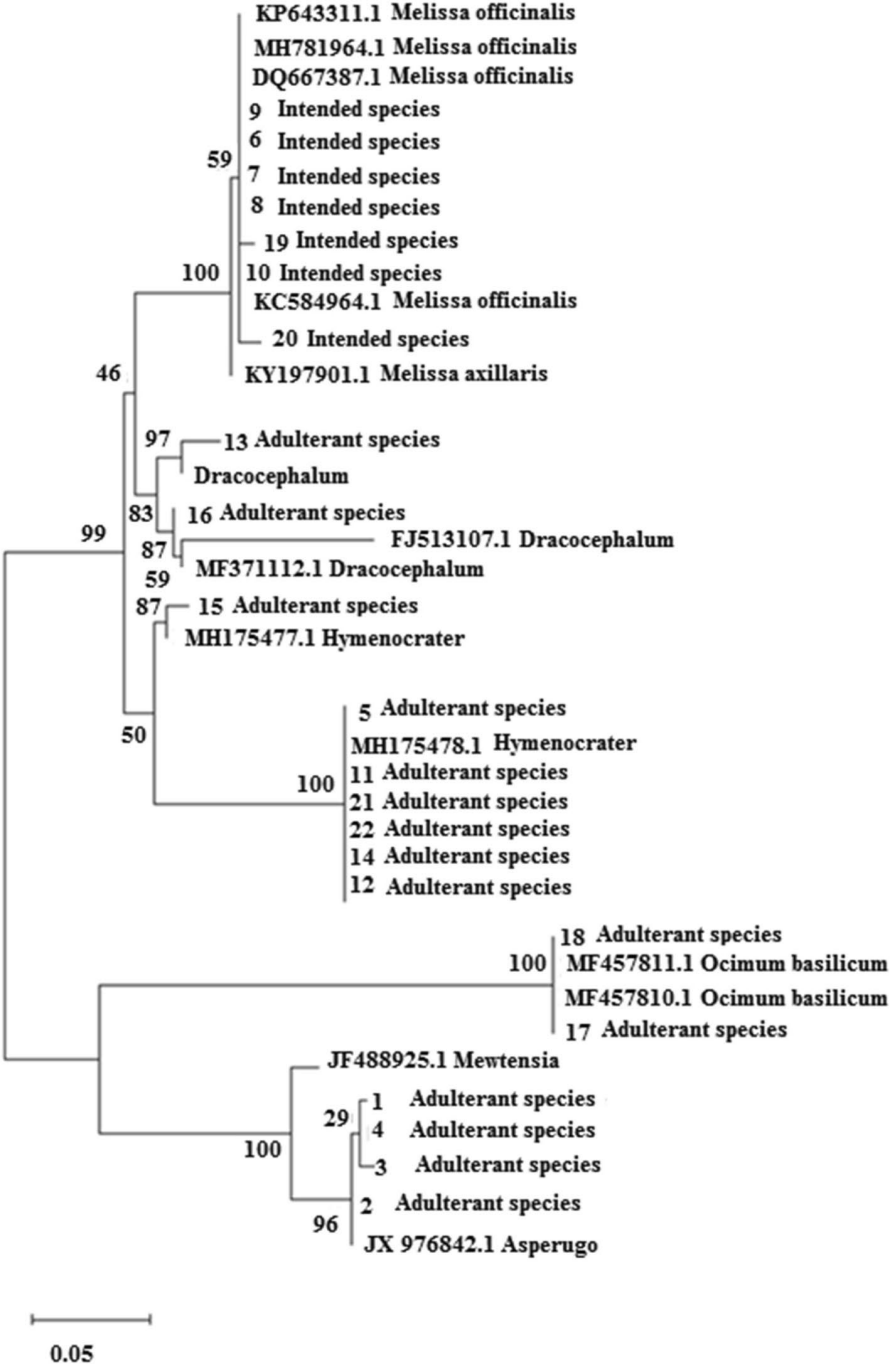
Maximum likelihood tree of the studied market products (1 to 22 based on Table 1) and reference species identified by BLAST query, based on trnH-psbA sequences. (Numbers above branches are bootstrap value)

Phylogenetic analyses of the market products/samples studied based on ITS and *trnH*-*psbA* regions were done. Phylogenetic analyses of the studied market products/samples were done based on ITS *trnH*-*psbA* regions. Different methods like Maximum parsimony, Maximum likelihood, and UPGMA produced similar results by MEGA software ver.7; therefore, only maximum likelihood plot is presented here (Figs. 7 and 8). In this plot, the market samples were placed next to the samples with the highest percentage of similarity in the BLAST (Table 4). For example, samples 1, 2, 3, and 4 that showed the highest similarity to *Asperugo procumbens* and *Mertensia virginica* were placed next to them (Table 4) showing the adulteration in marketing of this medicinal species.

## Discussion

In the current century, people face various kinds of threats such as food contamination, air pollution, diseases, and so on. Humans rely on different types of treatments to get healthier and better quality of life. Traditional medicine, mainly based on the prescription of the plants is considered as an important and historical treatment options in many countries. Therefore, the highest care and supervision should be carried out to maintain safe and real marketing of the medicinal plants and consumer products. In this regard, it is important to use the best and most effective modern techniques. For this purpose, the barcoding and molecular techniques must be used as the available tools [2].

Despite the high Medicinal importance of Lamiaceae, there are relatively few DNA barcodes available in this family [2]. Accurate and effective identification of gene sequences may vary in different plant species and should be investigated separately in each case. Has yielded relatively high levels of species resolution. The ITS and chloroplast gene region trnH-psbA has been proposed in many DNA barcode evaluation studies. For example, Laiou [26] investigated “core barcode” for land plants (*rbcL*, *matK*, and *trnH* - *psbA*) in 24 taxa. They sought to identify the right species based on the sequences, presence of DNA, gaps, and differentiation. The highest genetic diversity was observed in the *trnH* - *psbA* region; however, DNA barcoding was found in most cases using a sequence divergence in such a species. In general, species were successfully identified by 66.7%. Armenise [27] also reported successful barcoding in Pinaceae with *rbcL* + *trnH* - *psbA*.

In the present study, we showed that based on *ITS* and *trnH-psbA* sequence analysis *Melissa officinalis* samples were separated from *Hymenocrater*, *Dracocephalum*, and *Melissa axillaris* but these samples could not be separated from the others based on *trnL-F* sequences which indicate, this region was not suitable for DNA barcoding in *Melissa officinalis*. In addition, the same PCR bands were observed for the entire ITS and *trnH* - *psbA* gene region in our study therefore, we suggest that these region is a suitable barcode for the samples.

Adulteration of the medicinal plants has been reported throughout the world. For example, Newmaster [10] studied the plant product of a firm to determine the probability of cheating and succeeded in improving the DNA barcode in most plant products (91%) and all leaf samples (100%) with a resolution of 95% in the species. They found that, most of the studied products (59%) had DNA barcode of plant species that were not marked on the tag. Almost half of the products (48%) were confirmed through the analysis.

Sheidai [2] using ITS marker revealed that Kakoti (*ziziphora*) is adulterated with Thymus species in Iran. In this study, a low value of genetic distance within *Thymus* and *Ziziphora* samples (0.00–0.02) revealed a low degree of sequence variability within ITS sequences (high uniformity) whereas, a higher value of genetic distance was observed between *Ziziphora* and *Thymus* samples (0.10–0.20) that can consider as a barcode gap between the two groups. In the other study, Mohebi anabat [28] used *trnH-psbA* region to separate the Iranian saffron from the world’s saffron with a high genetic difference. We also reported that based on Kimura 2-parameters there was no genetic distance within *Melissa officinalis* (0.00) and a high value of genetic distance occurred between *Melissa officinalis* with other species (0.08 to 0.18).

In Iran, Badranjboye is one of the medicinal plants sold throughout the country. This study shows that this important product is introduced with the genera *Dracocephalum* and *Hymenocrater* as badranjboye. The results of morphology, odor, BLAST, phylogenetic tree, genetic distance, and barcode gap showed that at least 5 genera of *Hymenocrater*, *Dracocephalum*, *Asperugo*, *Mertensia*, *Trigonella*, and *Melilotus* were sold in the 22 markets studied as *Melissa Officinalis* which could have very negative effects on human health.

## Conclusion

Due to the medicinal value of *Melissa officinalis*, this species is sold in the Iranian markets, and some genera like *Dracocephalum*, *Hymenocrater*, *Asperugo*, *Mertensia*, *Trigonella*, and *Melilotus* have been sold instead of this species because of their similar name and increasing the weight of the product. Results of the present study showed the evidence of adulteration in *Melissa officinalis* products in the Iranian market and that ITS and *trnH*- *psbA* sequences are efficient molecular marker for barcoding of this medicinally important plant. Notably, to the best of our knowledge, this study is the first report on the *Melissa officinalis*.

## Data Availability

The datasets generated and/or analysed during the current study are not publicly available due [Plant materials were stored in Shahid beheshti university Herbarium. Since the samples collected from traditional herbal shops showed similarity to several species of the same genus, it is not possible to register them in the any database.] but are available from the corresponding author on reasonable request.

## Abbreviations

ITS: Internal transcribed spacer
*PsbA*–*trnH*: *Psba*–*trnH* intergenic spacer
DNA barcoding: Deoxyribonucleic acid barcoding
Cp-DNA: Chloroplast DNA
CTAB: Cetyltrimethyl- ammonium bromide 3 U
mM: Mill molar
dNTP: Deoxynucleoside triphosphate
NCBI: National Center for Biotechnology Information
MEGA: Molecular evolutionary genetics analysis
UPGMA: Unweighted paired group using average
BLAST: Basic Local Alignment Search Tool
rbcl: Ribulose bisphosphate carboxylase
